# CircZNF609 as a potential prognostic biomarker across multiple cancers: a systematic review and meta-analysis with ceRNA network exploration

**DOI:** 10.3389/fonc.2026.1933818

**Published:** 2026-08-28

**Authors:** Menglan Li, Sitong Tu, Kai Qian, Rong Chen, Liangnian Wei, Pengfei Li

**Affiliations:** 1 Department of Clinical Laboratory, Affiliated Hospital of Nanjing University of Chinese Medicine, Jiangsu Province Hospital of Chinese Medicine, Nanjing, Jiangsu, China; 2 College of First Clinical Medicine, Nanjing University of Chinese Medicine, Nanjing, Jiangsu, China

**Keywords:** cancer, ceRNA network, circZNF609, clinicopathological feature, meta-analysis, prognosis

## Abstract

**Background:**

CircZNF609 (hsa_circ_0000615), a back-spliced transcript derived from the ZNF609 gene locus, has garnered increasing attention for its functional roles in tumor progression across multiple cancer types. However, no pan-cancer meta-analysis has systematically evaluated its prognostic value and association with clinicopathological aggressiveness. We therefore performed this systematic review and meta-analysis to synthesize all available clinical evidence addressing this critical gap.

**Methods:**

We systematically retrieved literature from five databases (PubMed, Web of Science, Embase, the Cochrane Library and CNKI) up to April 2, 2026. Hazard ratios (HRs) and odds ratios (ORs) with 95% confidence intervals (CIs) were synthesized to quantify the link between circZNF609 levels and overall survival (OS) or clinicopathological variables. Heterogeneity assessment, sensitivity analysis, and publication bias evaluation were also performed. Additionally, a circZNF609-mediated ceRNA network was reconstructed based on experimentally validated targets.

**Results:**

A total of 12 studies involving 791 patients were included in this meta-analysis. Elevated circZNF609 expression was significantly associated with worse overall survival (HR = 2.31, 95% CI:1.74-3.07, *P* < 0.001; I² = 55.2%). Additionally, high circZNF609 expression was correlated with advanced TNM stage (OR = 2.34, 95% CI: 1.68-3.27, *P* < 0.001) and increased risk of lymph node metastasis (OR = 5.28, 95% CI: 3.48-8.00, *P* < 0.001). No significant associations were found between circZNF609 expression and age, sex, tumor size, or histological differentiation. Sensitivity analyses confirmed the robustness of the pooled results, and Begg’s test (*P* = 0.493) and Egger’s regression test (*P* = 0.197) indicated no significant publication bias. Mechanistically, the reconstructed ceRNA network suggested that circZNF609 may exert oncogenic effects via cell-cycle and metastasis-related pathways.

**Conclusions:**

Increased circZNF609 expression is associated with adverse survival outcomes and aggressive clinicopathological behavior across multiple malignancies. The ceRNA network offers mechanistic support for these clinical associations. CircZNF609 warrants validation in prospective, multi-ethnic cohorts as a promising prognostic biomarker and candidate therapeutic target.

## 1 Introduction

Cancer remains one of the leading causes of mortality worldwide and continues to impose a substantial healthcare burden despite continuous advances in diagnosis and treatment (1). During the past decade, molecularly targeted agents, immune checkpoint inhibitors, and individualized therapeutic strategies have improved outcomes in selected malignancies (2). Nevertheless, survival remains poor for many patients with advanced-stage disease, largely because of tumor heterogeneity, therapeutic resistance, and metastatic dissemination (3). Therefore, reliable biomarkers capable of predicting prognosis, monitoring disease progression, and guiding treatment selection are urgently needed.

Among non-coding RNAs, circular RNAs (circRNAs) are distinguished by their unique covalently closed circular structure, which protects them from degradation by exoribonucleases and endows them with significantly longer half-lives compared to their linear mRNA counterparts (4–6). Owing to tissue-specific expression and stable existence in body fluids, circRNAs have emerged as promising candidate diagnostic and prognostic tumor biomarkers (7, 8). Recent meta-analyses, including our previous work on circPVT1, further support the prognostic potential of oncogenic circRNAs across multiple solid tumors (9). Mechanistically, circRNAs exert biological functions via multiple canonical pathways: sponging endogenous miRNAs as competing endogenous RNAs, binding RNA-binding proteins as molecular scaffolds, modulating host gene transcription, and occasionally translating functional short peptides independent of cap structure (10, 11). In the field of oncology, dysregulated expression of circRNAs influences tumorigenesis, progression and metastatic potential by modulating core cellular processes including proliferation, apoptosis, invasion, metastasis and epithelial-mesenchymal transition (EMT) (12). Among these, the identification and mechanistic elucidation of metastasis-associated circRNAs have emerged as a cutting-edge direction in contemporary cancer molecular biology (13).

CircZNF609 (CircBase ID: hsa_circ_0000615) originates from exon 2 of the zinc-finger protein 609 locus on chromosome 15q22.31 and was first described in the context of myoblast proliferation, where it proved unusual in possessing an open reading frame spanning the back-splice junction (14). Work over the past several years has revealed that circZNF609 is abnormally expressed in various types of solid tumors. In most of these malignancies, including breast, bladder, ovarian, gastric, nasopharyngeal, lung, colorectal, biliary, laryngeal and glioma cancers, circZNF609 is upregulated and promotes cell proliferation, invasion and glycolysis via discrete miRNA/mRNA axes (e.g., miR-145-5p/p70S6K1, miR-1200/CDC25B, miR-324-5p/VDAC1, miR-483-3p/CDK6, miR-338-3p/HRAS) (15–19). Melanoma represents a notable exception: circZNF609 is decreased there and, by interacting with FMRP to destabilize RAC1 transcripts, actually restrains metastatic spread (20). This context-dependent duality underscores the biological complexity of circZNF609.

Several single-center studies have investigated the correlations between circZNF609 expression and patient survival or clinicopathological characteristics. Although accumulating studies have indicated the potential prognostic value of circZNF609, the existing evidence remains fragmented and inconclusive. Such limitations primarily stem from small sample sizes, heterogeneous cancer types, diverse cut-off values for stratifying circZNF609 expression levels, and inconsistent reported effect sizes across individual studies. To date, no quantitative synthesis has been performed to integrate these disparate findings and provide a comprehensive assessment of circZNF609’s prognostic utility across diverse malignancies. Accordingly, we conducted this systematic review and meta-analysis to pool all available data on the associations between circZNF609 expression, overall survival, and clinicopathological features. This study aims to clarify the generalizability of circZNF609 as a pan-cancer prognostic biomarker. In addition, we reconstructed a circZNF609-mediated ceRNA network using validated targets from the included clinical studies to provide mechanistic support for our pooled findings. As circZNF609 has been implicated in tumor immune regulation in preclinical research (10), the prognostic evidence established herein may also inform future exploration of its potential relevance to immunotherapy stratification.

## 2 Materials and methods

### 2.1 Literature retrieval

This study was conducted according to the Preferred Reporting Items for Systematic Reviews and Meta-Analyses (PRISMA 2020) guidelines (21). PubMed, Embase, Web of Science, the Cochrane Library, and CNKI were systematically searched from database inception to 2 April 2026. Search terms were listed as follows: (“circZNF609” OR “circular RNA ZNF609” OR “hsa_circ_0000615”) AND (“cancer” OR “tumor” OR “carcinoma” OR “neoplasm” OR “malignancy”) AND (“prognosis” OR “survival” OR “clinicopathological” OR “clinical outcome”). We also manually screened the reference lists of eligible studies and relevant reviews to identify additional eligible publications. Any discrepancies arising during the literature screening process were resolved through consensus discussion or consultation with a third reviewer.

### 2.2 Selection criteria

Studies were considered eligible if they met the following criteria: (i) enrolled patients with pathologically confirmed malignancy; (ii) quantified circZNF609 expression levels in tissue or body fluids; (iii) divided cohorts into high- and low-expression groups according to a stated threshold; and (iv) either presented HRs with 95% CIs for a time-to-event endpoint or supplied data permitting their reconstruction, and/or reported associations between circZNF609 expression and clinicopathological features.

We excluded duplicate publications, reviews, case reports, editorials, conference abstracts, purely cell-line or animal experiments lacking patient-level data, non-cancer studies, articles without usable survival or clinicopathological statistics, and retracted manuscripts.

### 2.3 Data extraction

Two investigators (Menglan Li and Liangnian Wei) independently populated a standardized form recording: first author, year, country, histological subtype, target miRNA, specimen type, cohort size, assay platform, expression threshold, group sizes, observation period, HR source, expression direction, survival endpoint and NOS score. Where HRs were unreported, we reconstructed them from Kaplan-Meier graphics via Engauge Digitizer v12.1 according to Tierney et al. (22). Clinicopathological data on age, sex, tumor size, histological grade, TNM stage, and lymph node metastasis (LNM) were likewise extracted. Conflicts were resolved by consensus or third-party adjudication.

### 2.4 Quality appraisal

Methodological rigor was scored on the Newcastle-Ottawa Scale (NOS), which rates cohort studies across selection (0-4), comparability (0-2) and outcome assessment (0-3). A total ≥ 6 denoted high quality (23). Both reviewers graded each study independently; discrepancies were resolved through discussion.

### 2.5 ceRNA network reconstruction and pathway enrichment

Experimentally confirmed circZNF609-miRNA-mRNA axes were extracted from the studies included in this meta-analysis. A consolidated network was assembled in Cytoscape 3.10.1 with circZNF609 as the seed, sponged miRNAs in the inner ring and validated downstream effectors in the outer ring. The resulting effector gene list was submitted to Metascape for hyper-geometric enrichment across Gene Ontology (GO) Biological Process and KEGG pathway ontologies; the human reference proteome served as background and Benjamini-Hochberg-adjusted *P* < 0.05 defined statistical significance.

### 2.6 Statistical methods

Analyses were executed in Stata SE 12.0 (StataCorp, College Station, TX). Pooled HRs with 95% CIs were calculated to assess the association between circZNF609 expression and OS, while pooled ORs were applied for clinicopathological feature comparisons. All HRs were standardized to represent the mortality risk in the high circZNF609 expression group relative to the low-expression group. Between-study variability was quantified by Cochran Q and the I² index. When Q yielded *P* < 0.10 or I² exceeded 50%, a random-effects model was adopted; otherwise, a fixed-effects model was utilized. Subgroup analyses were stratified by publication year, cohort size, follow-up duration, threshold criterion, HR derivation method, and methodological quality (NOS score). Robustness was tested by leave-one-out sensitivity analysis. Potential publication bias was evaluated using Begg’s rank correlation and Egger’s weighted regression tests, with *P* > 0.10 indicating no significant bias. All statistical tests were two-tailed, and *P* < 0.05 was considered statistically significant.

## 3 Results

### 3.1 Study selection and cohort overview

**Figure 1:**
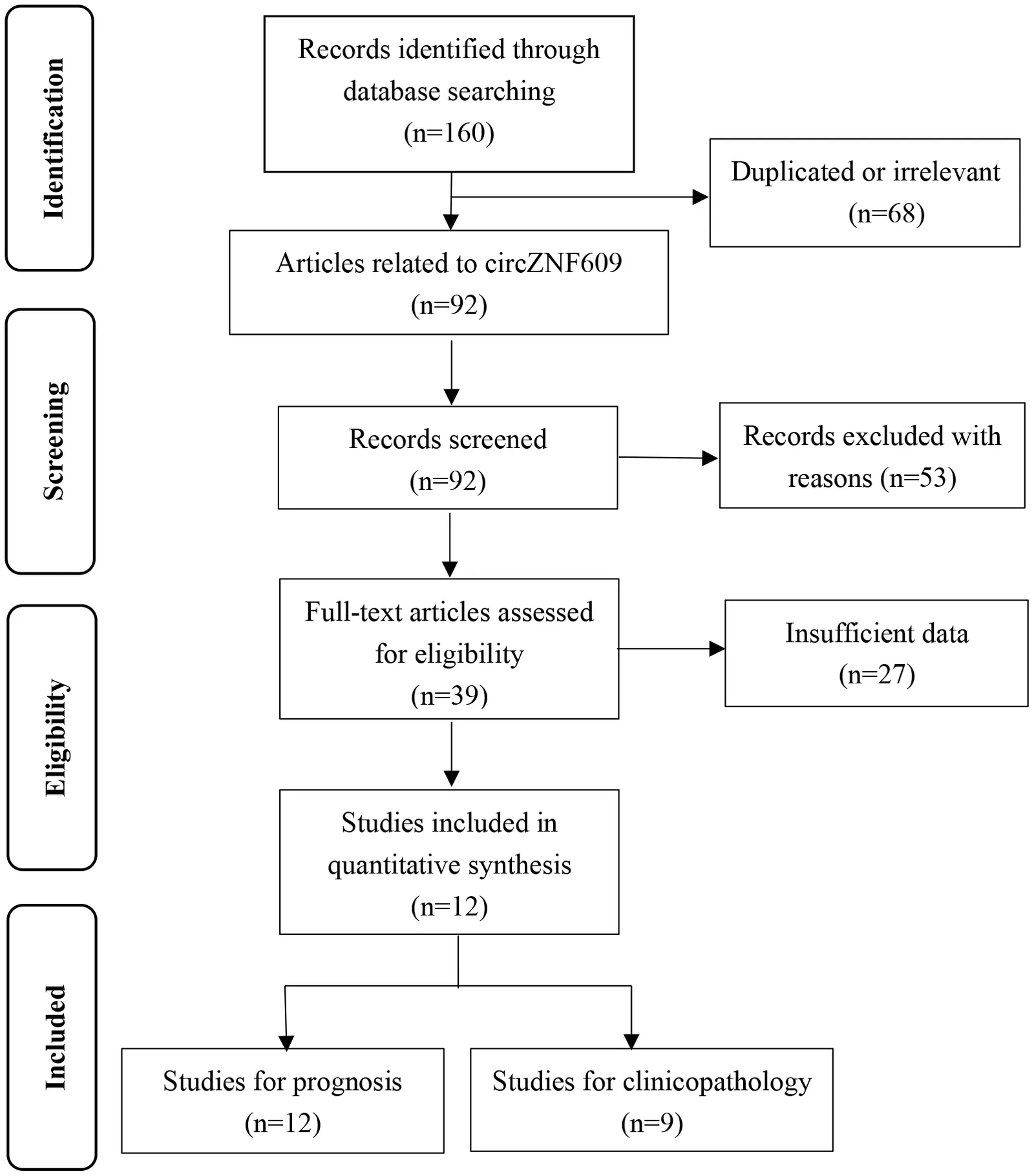
Flow chart of the process for study selection.

The initial literature search identified 160 potentially relevant records. After the removal of duplicates, 92 records remained for title and abstract screening. Of these, 53 records were excluded because they were unrelated to circZNF609, lacked clinical relevance, or represented reviews, editorials, and conference reports. Subsequently, 39 studies underwent full-text assessment. Twenty-seven of these articles were further excluded for the following reasons: insufficient survival information, absence of patient cohorts, unavailable HR data or publication retraction. Ultimately, 12 studies comprising a total of 791 patients met all inclusion criteria and were included in the final quantitative synthesis (**Figure 1**). The eligible studies, published between 2018 and 2024, were all conducted in China, with cohort sample sizes ranging from 37 to 143 patients. CircZNF609 expression was measured using quantitative real-time PCR (qRT-PCR). While most investigations analyzed in tumor tissues, one study specifically evaluated serum samples. Regarding the stratification strategy, eight studies utilized the median expression level as the cut-off value. Overall survival (OS) served as the principal endpoint in all included cohorts. Finally, the NOS scores ranged from 7 to 8, indicating high methodological quality across the selected studies. Detailed cohort characteristics are summarized in **Table 1**.

**Table 1:** Baseline characteristics of the 12 included studies.

Author	Year	Region	Cancer	miRNA	Sample	n	Method	Cut-off	Low	High	Follow-up time	HR source	Expression	Outcome	NOS
Feng et al. (16)	2023	China	BCa	miR-1200	Tissue	48	qRT-PCR	NA	21	27	60	KM	Up	OS	7
Ren et al. (17)	2024	China	OC	miR-324-5p	Tissue	40	qRT-PCR	Median	20	20	60	KM	Up	OS	8
Wu et al. (18)	2019	China	GC	miR-483-3p	Tissue	80	qRT-PCR	NA	NA	NA	60	Direct	Up	OS	7
Liu et al. (19)	2021	China	NPC	miR-338-3p	Tissue	60	qRT-PCR	NA	25	35	60	KM	Up	OS	8
Shang et al. (20)	2022	China	MM	NA	Tissue	37	qRT-PCR	Median	18	19	80	KM	Down	OS	7
Wang et al. (15)	2018	China	BC	miR-145-5p	Tissue	143	qRT-PCR	Median	72	71	120	KM	Up	OS	8
Wang et al. (24)	2021	China	NSCLC	miR-623	Tissue	62	qRT-PCR	Median	31	31	60	Direct	Up	OS	8
Yin et al. (25)	2022	China	LSCC	miR-134-5p	Tissue	42	qRT-PCR	Median	21	21	80	KM	Up	OS	8
Zhang et al. (26)	2020	China	CRC	NA	Serum	88	qRT-PCR	Median	43	45	60	KM	Up	OS	7
Li et al. (27)	2021	China	LUAD	NA	Tissue	68	qRT-PCR	NA	30	38	60	Direct	Up	OS	7
Du et al. (28)	2021	China	Glioma	miR-1224-3p	Tissue	62	qRT-PCR	Median	31	31	60	KM	Up	OS	8
Guan et al. (29)	2021	China	CCA	miR-432-5p	Tissue	61	qRT-PCR	Median	30	31	60	Direct	Up	OS	7

BCa, bladder cancer; OC, ovarian cancer; GC, gastric cancer; NPC, nasopharyngeal carcinoma; MM, melanoma; BC, breast cancer; NSCLC, non-small-cell lung cancer; LSCC, laryngeal squamous-cell carcinoma; CRC, colorectal cancer; LUAD, lung adenocarcinoma; CCA, cholangiocarcinoma; KM, Kaplan-Meier curve; OS, overall survival; NOS, Newcastle-Ottawa Scale; NA, cut-off method not reported.

### 3.2 CircZNF609 expression and overall survival

**Figure 2:**
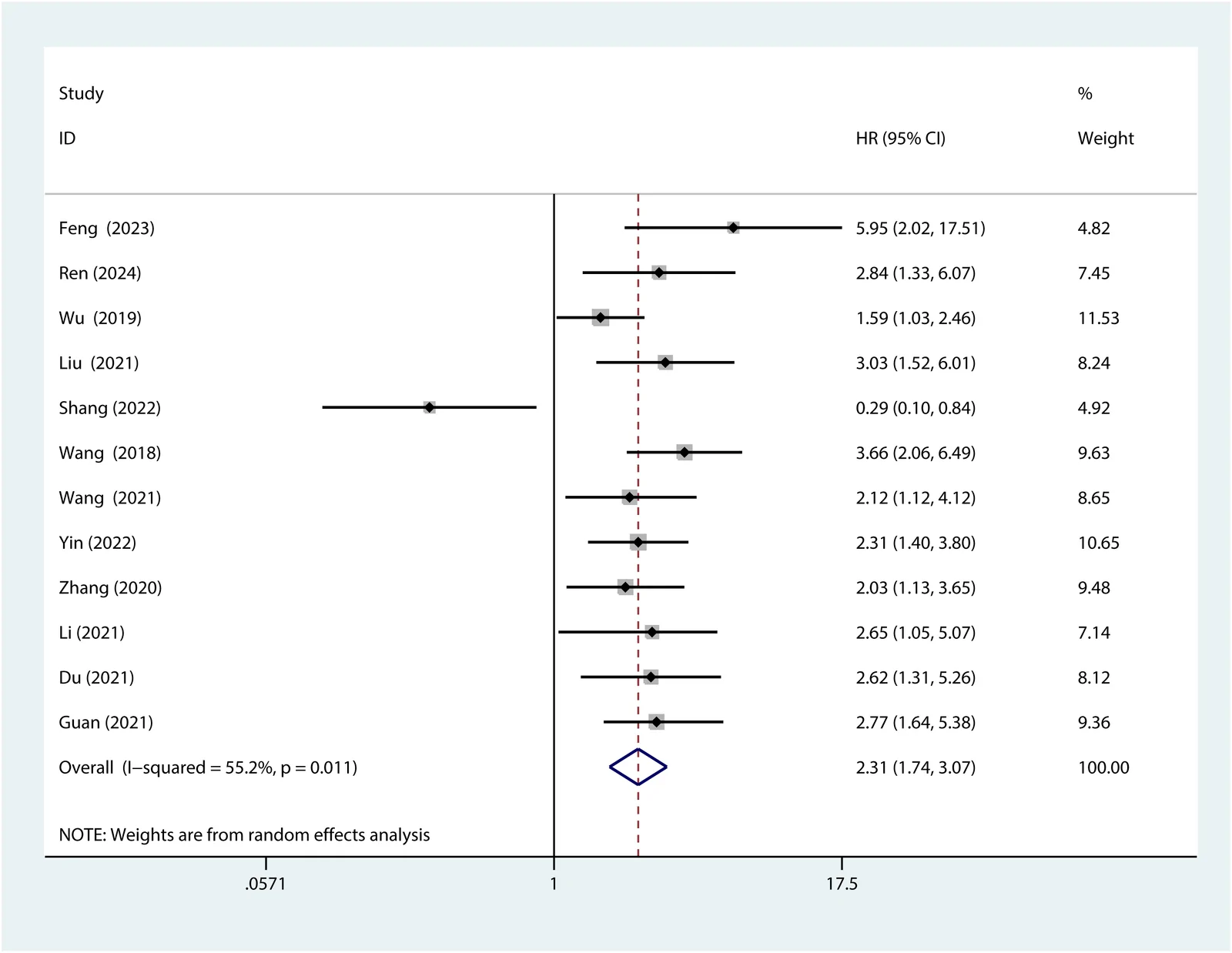
Forest plot of the pooled hazard ratios (HRs) for the association between circZNF609 expression and overall survival.

HR data for OS were extractable from all 12 included studies. Given the detection of moderate between-study heterogeneity (I² = 55.2%, *P* = 0.011), a random-effects model was applied. The pooled HR was 2.31 (95% CI: 1.74-3.07, *P* < 0.001), indicating that patients with elevated circZNF609 expression faced an approximately 2.3-fold increased mortality risk relative to those with low expression (**Figure 2**). Notably, the melanoma cohort represented the sole exception, exhibiting a protective prognostic effect (HR = 0.29) (20). This context-specific outlier constituted the primary source of the moderate inter-study heterogeneity across the pooled OS analysis.

### 3.3 Subgroup analyses for OS

**Figure 3:**
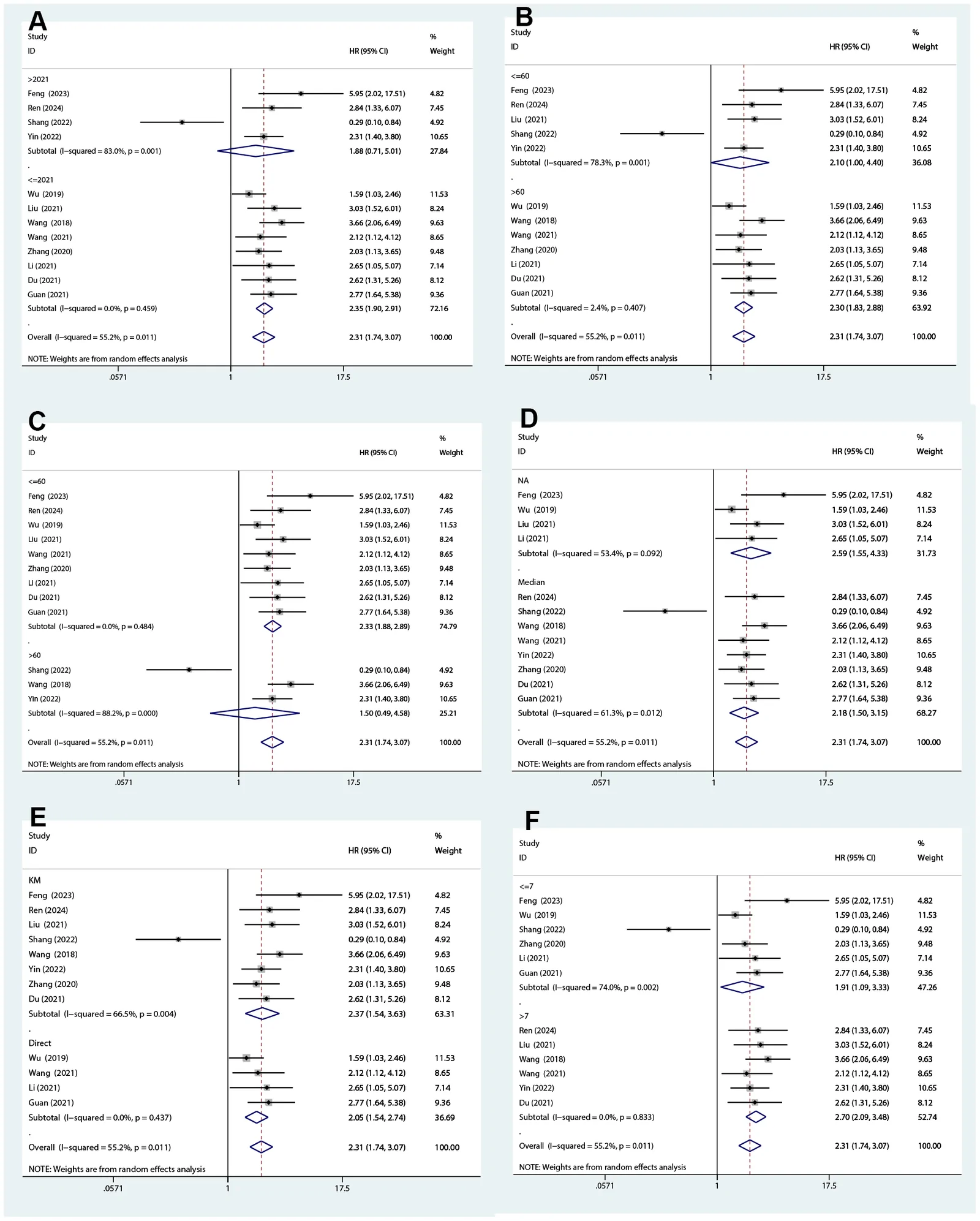
Forest plots of subgroup analyses for overall survival stratified by **(A)** publication year, **(B)** cohort size, **(C)** follow-up duration, **(D)** expression cut-off threshold, **(E)** HR derivation method, and **(F)** NOS score. *HR, hazard ratio; NOS, Newcastle-Ottawa Scale*.

To explore the sources of heterogeneity and verify the robustness of our findings, six stratified subgroup analyses were performed (**Table 2**; **Figures 3A–F**). Eight studies published before 2022 yielded a pooled HR of 2.35 (95% CI: 1.90-2.91) with no heterogeneity (I² = 0.00%), confirming a stable prognostic effect once the melanoma outlier and the most recently published studies were excluded. The seven larger cohorts (>60 patients) generated an almost homogeneous estimate (HR = 2.30, 95% CI: 1.83-2.88; I² = 2.4%), while smaller series (≤60 patients) exhibited wider confidence intervals and substantial heterogeneity (HR = 2.10, 95% CI: 1.00-4.40; I² = 78.3%). Studies with a follow-up duration of ≤60 months demonstrated no detectable heterogeneity (HR = 2.33, 95% CI: 1.88-2.89; I² = 0.0%). Notably, the NOS-stratified analysis was particularly informative: higher-quality reports (NOS > 7) produced a stronger and perfectly homogeneous estimate (HR = 2.70, 95% CI: 2.09-3.48; I² = 0.0%) compared with lower-scored studies (HR = 1.91, 95% CI: 1.09-3.33; I² = 74.0%).This observation indicates that higher methodological quality is associated with reduced between-study heterogeneity and more stable effect estimates, though confounding factors such as tumor type distribution also contribute to this pattern.

**Table 2:** Subgroup analyses of pooled hazard ratios for OS.

Subgroup	Studies (n)	OS		Heterogeneity	
		Pooled HR (95%CI)	*P* value	I^2^ (%)	*P* value
Publication year					
≤2021	8	2.35 (1.90-2.91)	<0.001	0.00	0.459
>2021	4	1.88 (0.71-5.01)	0.204	83.0	0.001
Sample size					
≤60	5	2.10 (1.00-4.40)	0.049	78.3	0.01
>60	7	2.30 (1.83-2.88)	<0.001	2.40	0.407
Follow up time					
≤60	9	2.33 (1.88-2.89)	<0.001	0.00	0.484
>60	3	1.50 (0.49-4.58)	0.480	88.2	<0.001
Cutoff value					
Median	8	2.18 (1.50-3.15)	<0.001	61.3	0.012
NA	4	2.59 (1.55-4.33)	<0.001	53.4	0.092
HRs source					
KM Curve	8	2.37 (1.54-3.63)	<0.001	66.5	0.04
Direct	4	2.05 (1.54-2.74)	<0.001	0.00	0.437
NOS					
≤7	6	1.91 (1.09-3.33)	0.023	74.0	0.002
>7	6	2.70 (2.09-3.48)	<0.001	0.00	0.833

### 3.4 Clinicopathological correlates

**Figure 4:**
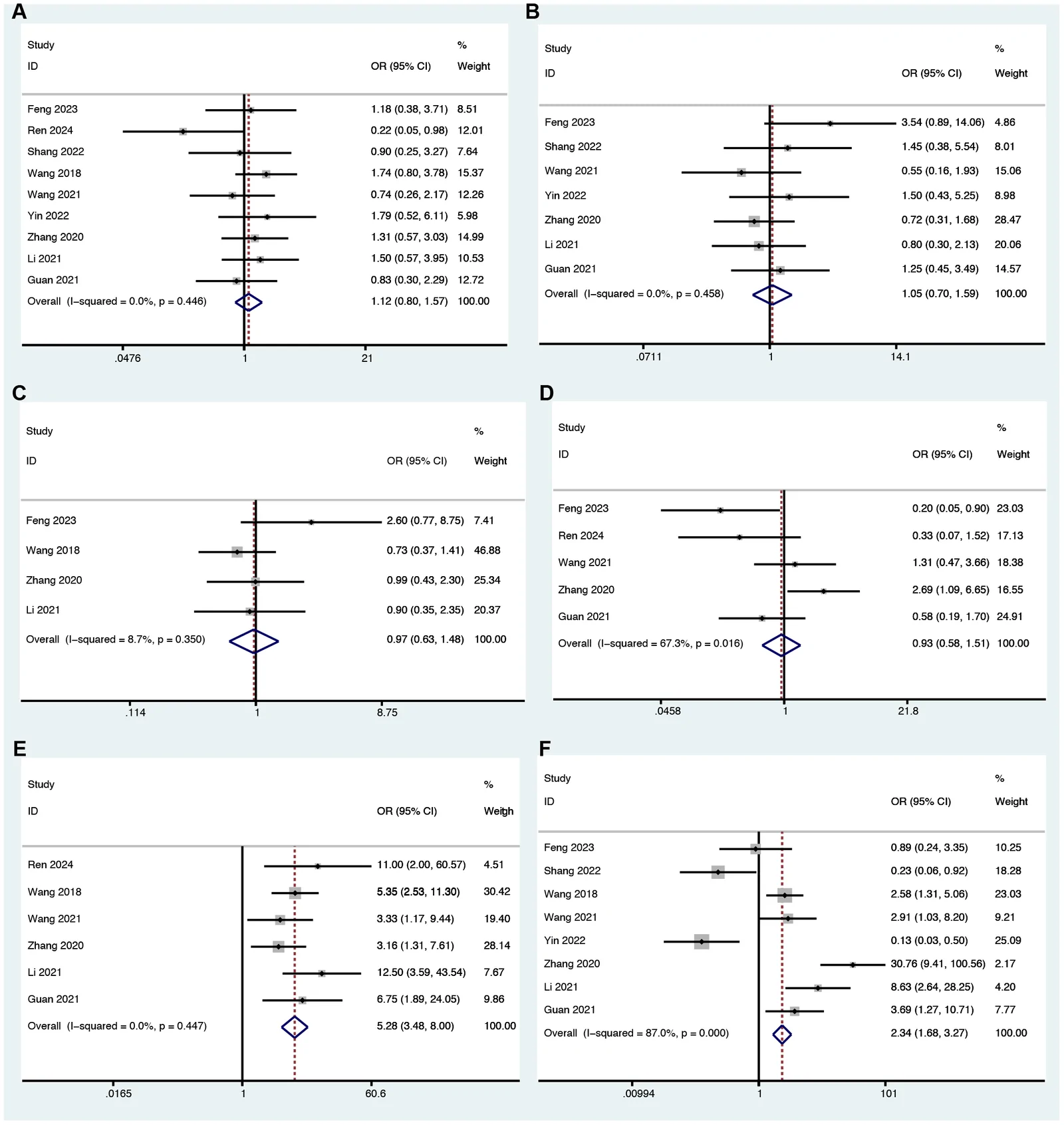
Forest plots evaluating the associations between circZNF609 expression and clinicopathological features: **(A)** age, **(B)** sex, **(C)** tumor size, **(D)** histological differentiation, **(E)** lymph node metastasis, and **(F)** TNM stage.

Six clinicopathological variables were amenable to pooled analysis. Elevated circZNF609 expression was strongly associated with an increased risk of lymph node metastasis (LNM)(6 studies; OR = 5.28, 95% CI: 3.48-8.00, *P* < 0.001; I² = 0.0%, fixed-effects; **Figure 4E**) and advanced TNM stage (8 studies; OR = 2.34, 95% CI: 1.68-3.27, *P* < 0.001; I² = 87.0%; random-effects; **Figure 4F**). However, the TNM-related result should be interpreted cautiously because the included studies involved different tumor types, for which TNM categories are not directly interchangeable. In contrast, no statistically significant associations emerged between circZNF609 and patient age (9 studies; OR = 1.12, 95% CI: 0.80-1.57; I² = 0.0%), sex (7 studies; OR = 1.05, 95% CI:0.70-1.59; I² = 0.0%), tumor size (4 studies; OR = 0.97, 95% CI:0.63-1.48; I² = 8.7%), or histological differentiation (5 studies; OR = 0.93, 95% CI:0.58-1.51; I² = 67.3%). Together, these results suggest that circZNF609 is more closely related to metastatic behavior than to tumor size or patient demographic factors.

### 3.5 Sensitivity analysis

**Figure 5:**
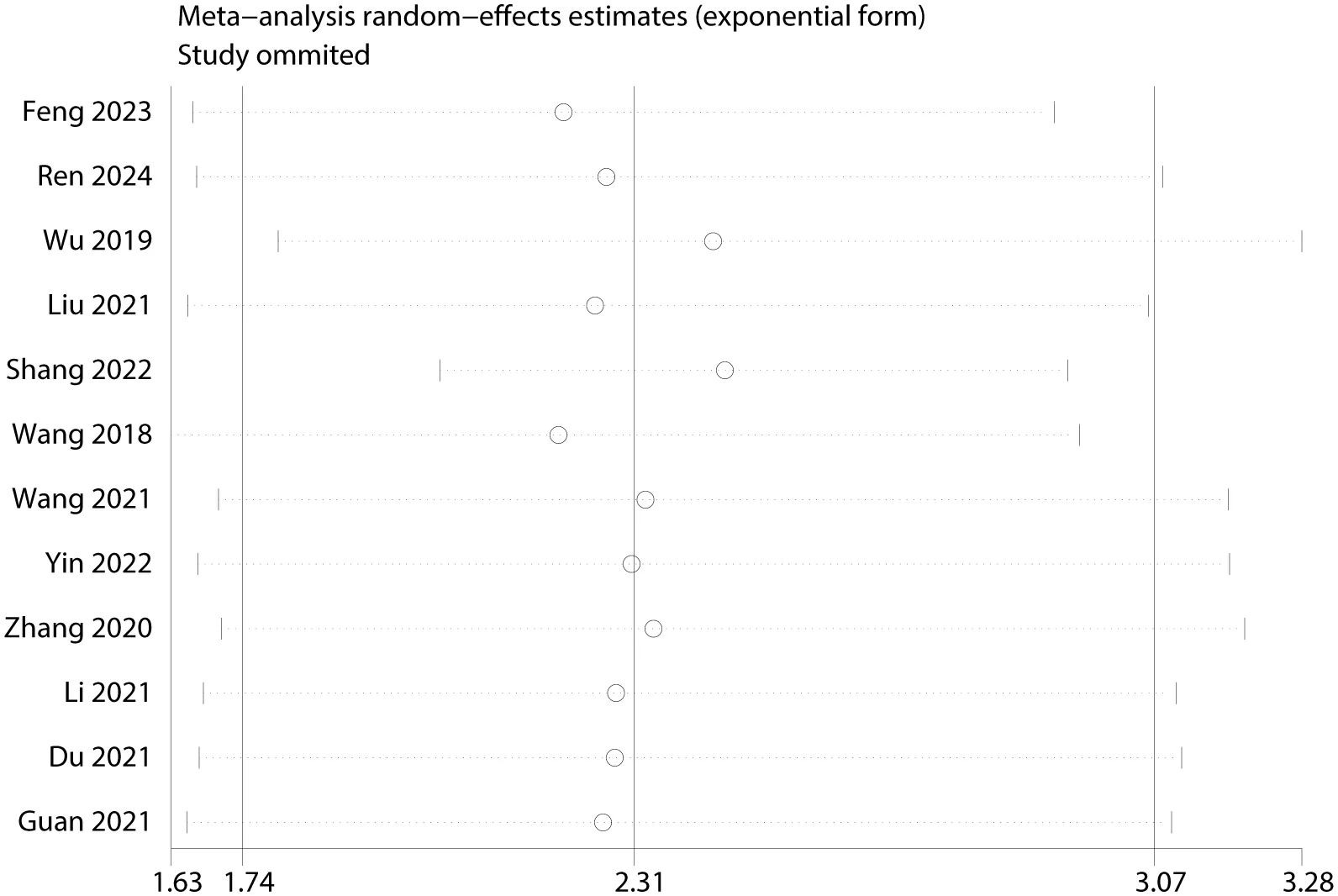
Leave-one-out sensitivity analysis for overall survival.

A leave-one-out sensitivity analysis, in which each study was sequentially removed, yielded recalculated pooled HRs ranging from 1.63 to 3.28. Importantly, all recalculated estimates remained statistically significant, confirming the robustness of the overall findings (**Figure 5**). As expected, the omission of the melanoma cohort shifted the point estimate modestly upward, which is consistent with its protective prognostic direction.

### 3.6 Publication bias

**Figure 6:**
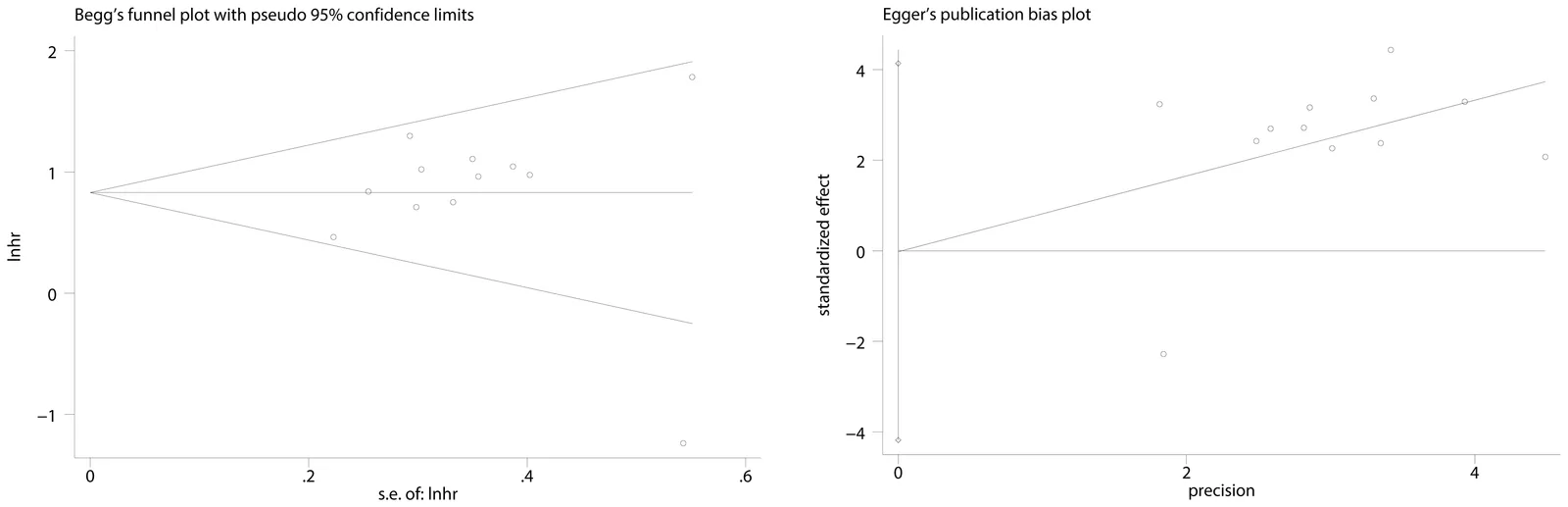
Begg’s funnel plot and Egger’s regression test for publication bias.

Potential publication bias was evaluated using both quantitative tests and visual inspection. Begg’s rank-correlation test (z = 0.69, *P* = 0.493; continuity-corrected z = 0.62, *P* = 0.537) and Egger’s weighted-regression test (t = 1.38, *P* = 0.197) did not identify statistically significant small-study effects. Furthermore, the corresponding funnel plot was visually symmetrical (**Figure 6**). These diagnostics collectively indicated no statistically detectable publication bias.

### 3.7 ceRNA network reconstruction and functional enrichment

**Figure 7:**
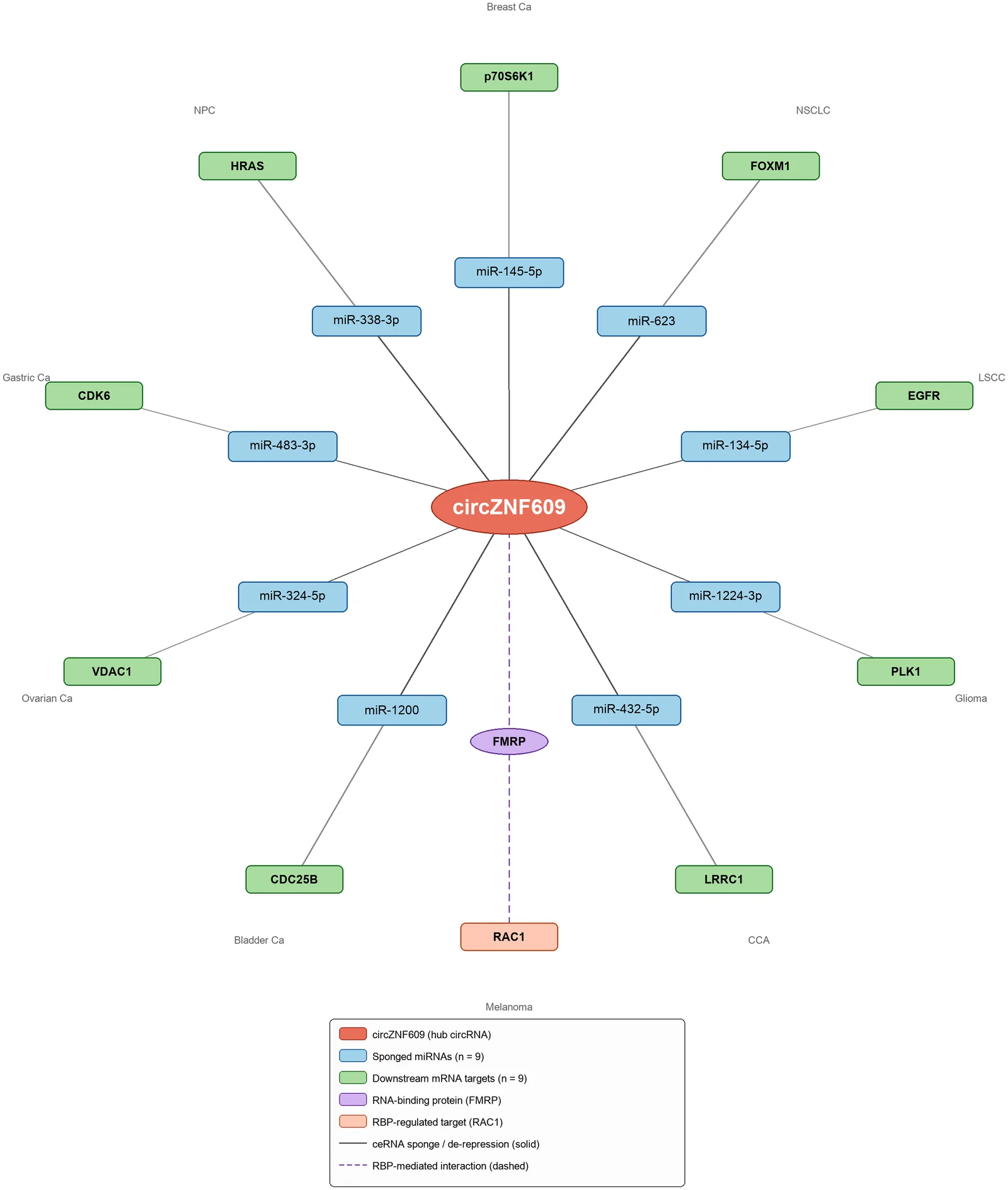
CircZNF609-mediated ceRNA network based on 12 included studies. The central orange node represents the host circZNF609, the blue inner ring comprises the sponged miRNAs (n = 9), and the green outer ring denotes the validated downstream protein-coding effector genes. The dashed edge highlights the non-canonical, miRNA-independent FMRP-RAC1 interaction observed in melanoma.

To explore the potential mechanism underlying the prognostic significance of circZNF609, experimentally validated circZNF609-associated regulatory axes from the included studies were integrated. As shown in **Figure 7**, nine miRNA targets of circZNF609 were identified, including miR-1200, miR-324-5p, miR-483-3p, miR-338-3p, miR-145-5p, miR-623, miR-134-5p, miR-1224-3p, and miR-432-5p. Their downstream effectors included cell-cycle regulators and oncogenic signaling molecules, including CDC25B, CDK6, PLK1, FOXM1, HRAS, p70S6K1, EGFR, alongside VDAC1 and LRRC1. In addition, a non-canonical FMRP-RAC1 interaction was identified in melanoma, suggesting a context-dependent regulatory pattern of circZNF609.

**Figure 8:**
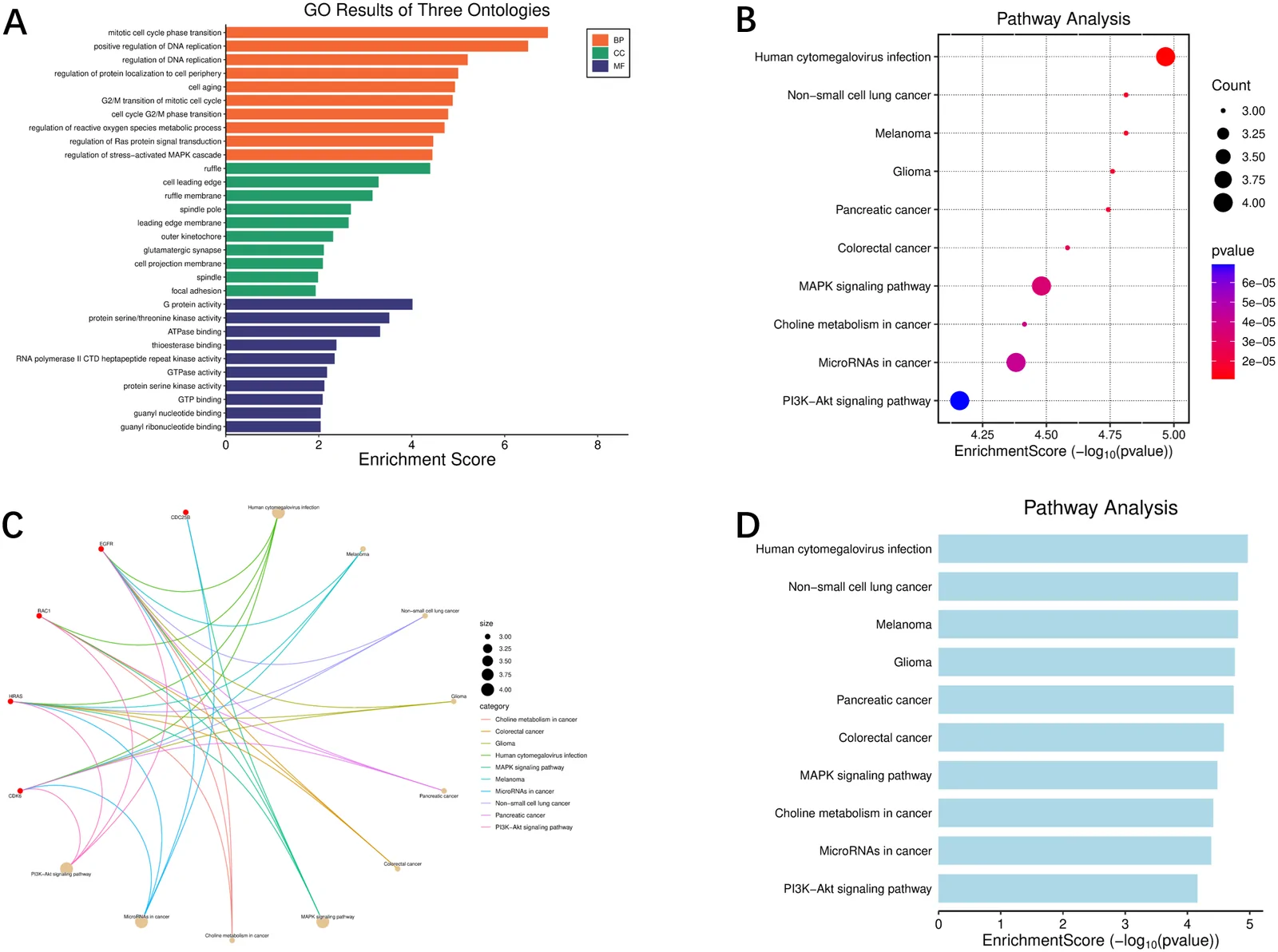
Functional enrichment analysis of circZNF609-regulated downstream effectors: **(A)** GO Biological Process. **(B–D)** KEGG pathway.

GO enrichment analysis showed that these downstream targets were mainly associated with cell-cycle regulation, DNA replication, cytoskeletal remodeling, and kinase/GTPase-related signal transduction. KEGG analysis further indicated enrichment in cancer-related pathways, particularly the MAPK signaling pathway, PI3K-Akt signaling pathway, and microRNAs in cancer. Overall, these results suggest that circZNF609-associated targets may participate in tumor progression mainly by regulating cell-cycle activity, DNA replication, cell motility, and MAPK/PI3K-Akt-related oncogenic signaling. (**Figures 8A–D**).

## 4 Discussion

To the best of our knowledge, this is the first systematic review and meta-analysis to quantitatively evaluate the prognostic and clinicopathological significance of circZNF609 across multiple cancers. By pooling data from 12 eligible studies encompassing a total of 791 patients, we demonstrated that elevated circZNF609 expression is significantly associated with poor OS. Furthermore, high circZNF609 expression strongly correlated with advanced TNM stage and LNM, but not with age, sex, tumor size, or histological differentiation. This pattern suggests that circZNF609 may be more closely associated with aggressive and metastatic tumor behavior than localized tumor growth.

The biological plausibility of these clinical observations is supported by the regulatory functions of circZNF609, predominantly through ceRNA networks (30). In our reconstructed network, circZNF609 sequesters a diverse array of tumor-suppressive miRNAs (e.g., miR-145-5p, miR-338-3p), thereby derepressing downstream effectors such as p70S6K1, FOXM1, EGFR, PLK1, LRRC1, CDC25B, VDAC1, CDK6, and HRAS. These targets can be functionally grouped into two interconnected modules involving cell-cycle progression and metastatic invasion, providing a biologically plausible framework for the associations between circZNF609 dysregulation and the aggressive clinical phenotypes observed in the pooled analysis.

The ceRNA network and enrichment analyses further suggest that circZNF609 may promote malignant progression through the coordinated regulation of cell-cycle activity, oncogenic signaling, and cellular motility. Several downstream targets, including CDC25B, CDK6, PLK1, FOXM1, EGFR, HRAS, and p70S6K1, are closely related to cell-cycle transition and MAPK/PI3K-Akt-related signaling. This is consistent with our GO enrichment results highlighting cell-cycle regulation and DNA replication, as well as KEGG enrichment of MAPK and PI3K-Akt signaling pathways (31). Among the included studies, circZNF609 was shown to promote bladder cancer progression through the miR-1200/CDC25B axis, gastric cancer proliferation and migration through the miR-483-3p/CDK6 axis, glioma progression through the miR-1224-3p/PLK1 axis, and breast cancer growth, migration, and invasion through the miR-145-5p/p70S6K1 axis (15, 16, 18, 28). These findings indicate that circZNF609 may function as an upstream ceRNA regulator that releases multiple oncogenic effectors from miRNA-mediated repression. This interpretation is further supported by independent mechanistic studies outside the included meta-analysis. For example, Liu et al. demonstrated that circZNF609 acts as a miRNA sponge to antagonize miR-142-3p-mediated repression of GNB2, which suppresses cell proliferation and migration and induces cell apoptosis (32). Nevertheless, because the enrichment analysis was based on a relatively small set of experimentally validated downstream genes, these pathway-level findings should be regarded as hypothesis-generating rather than definitive mechanistic evidence.

The association between circZNF609 and metastatic potential is also supported by both our pooled clinicopathological findings and experimental evidence. In this meta-analysis, high circZNF609 expression was strongly associated with LNM, whereas no significant association was observed with tumor size. This pattern suggests that circZNF609 may be more closely linked to invasive behavior than to local tumor expansion alone (33). Consistently, GO enrichment identified motility-related terms such as cell leading edge, ruffle formation, focal adhesion, and GTPase-related signal transduction. Several included studies support this interpretation: circZNF609 promoted migration or invasion through the miR-338-3p/HRAS axis in nasopharyngeal carcinoma, the miR-134-5p/EGFR axis in laryngeal squamous cell carcinoma, and the miR-432-5p/LRRC1 axis in cholangiocarcinoma (19, 25, 29). Similar findings have been reported in studies outside the meta-analysis. For example, circZNF609 has been reported to promote colorectal cancer migration through the miR-150/Gli1 axis (34) and enhance nasopharyngeal carcinoma growth and metastasis through the miR-150-5p/Sp1 axis (35). More importantly, circZNF609 was shown to interact with CKAP5 mRNA and regulate microtubule dynamics and tumorigenicity (36), providing direct mechanistic support for the cytoskeleton-related enrichment observed in our analysis. Taken together, these findings suggest that circZNF609 may facilitate metastatic dissemination by integrating ceRNA-mediated oncogenic signaling with cytoskeletal remodeling and cell motility (37). Notably, although circZNF609 is well-characterized for its protein-coding capacity during myogenic differentiation (14), the oncological studies incorporated in our meta-analysis have largely centered on its function as a ceRNA. Future studies are needed to determine whether its encoded peptides also contribute to tumor progression.

Interestingly, our meta-analysis also highlights the tissue-specific duality of circZNF609. Although it acts as an oncogenic regulator in most evaluated malignancies, including breast, gastric, and bladder cancers, it appears to exert a tumor-suppressive effect in melanoma. Shang et al. reported that circZNF609 is downregulated in melanoma and restricts metastatic spread through a miRNA-independent mechanism, specifically by interacting with the RNA-binding protein FMRP to destabilize RAC1 transcripts (20). This context-dependent behavior is a recognized feature of certain non-coding RNAs, whose ultimate function is dictated by the specific repertoire of miRNAs, RNA-binding proteins, and downstream effectors available in a given cellular microenvironment (6, 38). For example, circHIPK3 acts as an oncogene in liver and colorectal cancers by sponging the tumor-suppressive miR-124, whereas it functions as a tumor suppressor in bladder cancer by sequestering the oncogenic miR-558 to inhibit metastasis (39, 40). Although the melanoma cohort contributed substantially to inter-study heterogeneity, all studies compared relatively high versus low circZNF609 expression within the same tumor type, allowing the HRs to be pooled. Moreover, exclusion of the melanoma study did not materially alter the pooled estimate, supporting the stability of the overall association.

Beyond its roles in tumor cell-intrinsic aggressiveness, circZNF609 may also influence the tumor microenvironment and immune response, which could further modulate patient prognosis. A recent bladder cancer study reported that circZNF609 facilitates immune escape and reduces immunotherapy sensitivity via the IGF2BP2/CD36 axis (41). More broadly, circular RNAs broadly are recognized regulators of tumor immunity (10). Although our meta-analysis did not evaluate immune-related endpoints, the prognostic association identified herein provides a rationale for future studies to explore circZNF609 as a candidate immunotherapy stratification biomarker.

From a translational perspective, circZNF609 exhibits biological features that support its potential as a liquid biopsy candidate (42). Its covalently closed circular structure confers resistance to exonuclease degradation in blood, and one included study has demonstrated that circZNF609 can be reliably detected in cell-free serum (26). Exosome-derived circRNAs are further protected from plasma RNase activity, and exosomal circRNAs such as circSATB2 have been reported to mediate intercellular communication in the tumor microenvironment in non-small cell lung cancer (43–45). Given the strong association between circZNF609 and lymph node metastasis, circZNF609 may be a potential blood-based marker for monitoring metastatic risk during postoperative follow-up, but its clinical utility remains to be validated in large prospective cohorts. More sensitive methods, such as droplet digital PCR or nanopore sequencing, could further improve the detection of low-abundance circular transcripts in future studies.

Moderate heterogeneity was observed across the included studies (I² = 55.2%), mainly due to the opposite prognostic effect reported in the melanoma cohort; excluding this cohort yielded a pooled HR of 2.44 (95% CI: 2.03–2.94, P < 0.001) with I² = 0.0%. Subgroup analyses further suggested that smaller sample sizes (≤60 patients), HRs extracted from Kaplan–Meier curves, longer follow-up (>60 months), and lower study quality may contribute to variability among studies. For TNM stage analysis, the high heterogeneity (I² = 87.0%) warrants cautious interpretation. This variability may be explained by two factors. First, TNM staging is not fully comparable across different tumor types; for example, stage III disease does not necessarily carry the same biological meaning in breast cancer and cholangiocarcinoma (46). Second, the opposite prognostic trend in the melanoma subgroup may have further increased the pooled heterogeneity. Because the number of studies for each tumor type was limited, tumor-specific meta-regression could not be performed to clarify this issue. With more tumor-specific data in the future, stratified analyses will be better able to address this limitation (47).

Despite these consistent findings, several limitations warrant careful consideration. First, all included studies were conducted in Chinese populations. Therefore, whether these results can be generalized to other ethnic groups, environmental backgrounds, and healthcare settings remains uncertain. Second, all cohorts were retrospective and had relatively small sample sizes (37–143 patients), which limited adjustment for important clinical confounders such as performance status, comorbidities, and treatment regimens. Furthermore, the survival analysis was limited to OS because data on other endpoints, such as disease-free survival and progression-free survival, were insufficient. Third, methodological differences across studies may have affected the results. At present, there is no agreed standard for circZNF609 detection or for defining high and low expression. Additionally, hazard ratios in 8 of the 12 studies had to be extrapolated from published Kaplan-Meier curves, introducing unavoidable digital extraction variance. Finally, although Begg’s and Egger’s tests indicated no significant publication bias, their statistical power is inherently constrained by the small number of included studies. Thus, the possibility of unpublished negative studies cannot be completely excluded.

Further work is needed before circZNF609 can be moved toward clinical use. First, multicenter, prospective, and multi-ethnic cohort studies are required to define reliable prognostic cut-off values and to determine whether circZNF609 provides additional prognostic information beyond conventional TNM staging. Second, rigorously standardized pre-analytical and analytical protocols for circZNF609 detection (including tissue fixation, RNA isolation, and expression cut-off values) should be established to improve inter-laboratory reproducibility. Third, the diverse molecular mechanisms of circZNF609 across tumor types should be further elucidated, with particular emphasis on the biological functions of its encoded peptides and its spatial expression patterns within tumors. Fourth, the clinical utility of circZNF609 as a liquid biopsy biomarker for dynamic post-operative monitoring and early detection of metastatic risk should be prospectively evaluated.

## 5 Conclusion

This study highlights elevated circZNF609 as a promising prognostic biomarker that significantly correlates with poor overall survival and advanced clinicopathological features across multiple cancers. The reconstructed ceRNA network provides a possible mechanistic explanation, linking circZNF609-mediated miRNA regulation to tumor aggressiveness and metastasis-related phenotypes. Although circZNF609 showed stable prognostic performance in this meta-analysis, its clinical application will require standardized detection methods and further validation in prospective, multi-ethnic cohorts. Future studies should also explore the potential roles of circZNF609 in tumor immune regulation and immunotherapy response stratification to extend its clinical utility.

## Data Availability

The original contributions presented in the study are included in the article/supplementary material. Further inquiries can be directed to the corresponding authors.
